# Open-Source Strain Gauge System for Monitoring Pressure Distribution of Runner’s Feet

**DOI:** 10.3390/s23042323

**Published:** 2023-02-19

**Authors:** Klaudia Kromołowska, Krzysztof Kluza, Eliasz Kańtoch, Piotr Sulikowski

**Affiliations:** 1 AGH University of Science and Technology, al. A. Mickiewicza 30, 30-059 Krakow, Poland; 2 Faculty of Computer Science and Telecommunications, Cracow University of Technology, ul. Warszawska 24, 31-155 Kraków, Poland; 3 Faculty of Computer Science and Information Technology, West Pomeranian University of Technology, ul. Żołnierska 49, 71-210 Szczecin, Poland

**Keywords:** foot pressure distribution, shoe inserts mobile application, smart shoe inserts, smart shoe insoles, insole sensors

## Abstract

The objective of the research presented in this paper was to provide a novel open-source strain gauge system that shall enable the measurement of the pressure of a runner’s feet on the ground and the presentation of the results of that measurement to the user. The system based on electronic shoe inserts with 16 built-in pressure sensors laminated in a transparent film was created, consisting of two parts: a mobile application and a wearable device. The developed system provides a number of advantages in comparison with existing solutions, including no need for calibration, an accurate and frequent measurement of pressure distribution, placement of electronics on the outside of a shoe, low cost, and an open-source approach to encourage enhancements and open collaboration.

## 1. Introduction

The development of wearable sensor technology and the miniaturization of electronic systems opens new possibilities for improving patient monitoring. One of the popular applications of wearable sensors is to monitor human movement, especially the number of steps taken. According to the World Health Organization, an average person should take from six to eight thousand steps a day [1], which is about 2.5 million steps a year.

The development of innovative solutions for foot movement monitoring can support the process of early detection and the treatment of musculoskeletal disorders, which are often caused by foot disorders [2]. Foot deformation, inadequate rotation, or improper balance exerts an influence on all elements of the movement apparatus: knees, hips, and, over time, the spine, and, thus also, the whole body. The early detection of this problem can be achieved by tracking every single movement of a person. However, the process of monitoring feet movement and pressure is challenging because of sophisticated and costly equipment. Moreover, by detecting abnormalities in children’s movement, people would be able to eliminate health problems long before the first symptoms (e.g., early detection and correction of the pressure of a young child’s feet can prevent an unwanted curvature of the spine).

In this paper, we address this gap by exploring an accessible and easy-to-use in sole-based wearable pressure monitoring system designed with off-the-shelf components for everyday running training.

**Figure 1:**
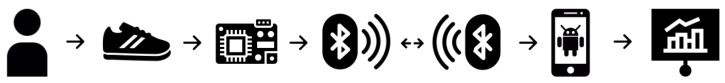
System operation diagram.

The system for precise monitoring of feet movement patterns would be a helpful tool not only for physiotherapists or qualified trainers but also for a person who exercises to control their posture while running. Therefore, our approach is focused on a wearable solution applicable to daily activities. The schematic overview of the operation of our system is presented in Figure 1. The data from the insole goes through the microcontroller software and is transferred to our mobile application for visualization.

The aim of the paper is to introduce an open-source strain gauge system, which we designed, implemented, and validated to allow runners to monitor their feet’s pressure distribution. We have provided the literature overview of the existing approaches to processing strain gauge signals taken from human feet and described the implementation of the application to display measurements in a simple form on a mobile device.

The paper is structured as follows. In Section 2, we present a brief overview of the existing related works. Section 3 presents the materials and methods for our project, especially focusing on the system architecture along with the hardware and software designs. Our results are described in Section 4, where we present the working system, its testing and validation. Section 5 discusses the applicability of the proposed solution. We conclude the paper with a summary and future works in Section 6.

## 2. Related Works

In this section, we compare and contrast our project with related works and highlight the main advantages and novelty of this study. A general comparison of developed solutions with existing insole-based sensor systems used for gait and activity monitoring is presented in Table 1.

Insole-based systems for health monitoring have been increasingly popular recently [3,4]. Moreover, there is a market demand for low-cost, unobtrusive, and reliable wearable healthcare solutions [5]. In fact, the data generated by these devices might also be used for many different purposes, such as movement classification [6,7], slip detection [8,9,10], or fall risk assessment [11,12].

In a recent study [3], Sophini Subramaniam et al. presented a survey on insole-based lower-limb health monitoring systems. The authors found that the most commonly measured parameters associated with lower-limb health were foot plantar pressure, temperature, pulse rate, and gait dynamics. The authors point out that telemonitoring of those parameters is important, especially for older people. The authors conclude that current challenges include but are not limited to sensors and their placements, ensuring user comfort and ease of use, power efficiency, and data privacy.

In the case of problems with an abnormal body posture, chronic pain in the lower limbs that worsens while walking, recurrent injuries, or conditions after surgery, the doctor’s common recommendation would be baropodometry [13], which is a computerized foot test performed while standing or walking. The test consists of measuring the forces of the ground reaction to the pressure of the patient’s feet. There are mats available up to a length of about two meters; consequently, in practice, this test does not allow measurement while running when human steps are much longer (stride length during running increases by about a half compared to walking [14]). However, performing such a test, e.g., several times a month might be a significant burden for patients due to the cost of up to USD 40 [13] for each trial. Additionally, it should be taken into account that, for athletes, the way the feet are placed (and thus the results of the test) will vary significantly after strenuous exercise and at the end of a light workout, which does not significantly affect muscle fatigue.

**Table 1:** General comparison of the developed solution with existing insole-based sensor systems used for gait and activity monitoring.

Author(s)	Application	Sensor Types	Number of Sensors	Wireless Data Transmission	Pressure Range	Battery Lifetime	Calibration Required	Transmission Distance	Real Time Monitoring	Cost	Opensource
Saito et al. [15]	plantar pressure graphs at mobile personal computer	pressure-Sensitive Conductive Rubber	7	yes	25–150 kPa	20 h	yes	10 m (Bluetooth)	N/A	high (USD 1000)	N/A
Sorrentino et al. [16]	online visualization tool	capacitive pressure sensors	336 (280 pressure, 56 temperature)	no	at least 90 kPa	-	yes (only once)	-	yes	low (<500 EUR)	no
Lin et al. [17]	gait monitoring graphs (Voltage to Time)	triboelectric sensor	2	no	at least 400 kPa	-	N/A	-	yes	high	no
Deng et al. [18]	plantar pressure monitoring	piezoelectric nanogenerators, triboelectric-electromagnetic nanogenerator	32	yes	up to 200 kPa	N/A	N/A	10 m (Bluetooth)	yes	low	no
Jung et al. [19]	no	pneumatic pressure sensors and IMU	5	yes	N/A	10 h	N/A	N/A	yes	high	no
Mustufa et al. [20]	no	piezoelectric sensors, IMU, temperature sensor, force sensor	35	yes	up to 1000 kPa	120 min (280 mAh)	N/A	10 m (Bluetooth)	yes	low	no
Jagos et al. [21]	applications are being implemented	resistive pressure sensors and IMU	5	yes	up to 50 kPa	small (150 mAh)	N/A	30 m (Bluetooth 2.1+)	no	N/A	no
Roth et al. [22]	custom android app	resistive pressure sensors and IMU	4	yes	up to 1000 kPa	40 h	N/A	15–30 m (BLE)	N/A	N/A	no
Djamaa et al. [23]	desktop and android app (visualize the pressure sensors data as heat-map)	resistive pressure sensors, resistive bend sensors, IMU	5	yes	N/A	N/A	yes	15–30 m (BLE)	yes	low	no
Farid et al. [24]	use FeetMe Evaluation mobile application	pressure sensors and IMU	19	yes	N/A	small (110 mAh)	yes	15–30 m (BLE)	yes	N/A	no
Duong et al. [25]	N/A	resistive sensors and IMU	9	yes	N/A	N/A	yes	40 m (Wi-Fi)	N/A	N/A	no
Chen et al. [26]	plantar pressure on the smartphone application	resistive pressure sensors and IMU	97	yes	N/A	1000 mAh	N/A	10 m (Bluetooth)	N/A	N/A	no
Lou et al. [27]	N/A	piezoresistive pressure sensors	14	yes	up to 800 kPa	N/A	N/A	up to 150 m	yes	N/A	no
Chadel et al. [28]	no, data collected at SD card	piezoelectric pressure sensors and IMU	6	yes	N/A	N/A	no	40 m (Wi-Fi)	no	low	no
Martini et al. [29]	N/A	optoelectronic pressure sensors	16	yes	N/A	N/A	no	∼35 m	yes	N/A	no
This project	dedicated mobile application	resistive pressure sensors	16	yes	∼400 kPa	min. 72 h	no	10 m (Bluetooth)	yes	150 EUR	yes

One of the solutions for smart insoles with a mobile application is Digitsole [30]. According to the manufacturer’s description, the insoles have all important functionalities—they measure foot balance, step length and stability, support zones, detect pronation/supination, and monitor flight time and contact time. Built-in GPS ensures convenient viewing of statistics. In addition, the manufacturer offers personalized support and many tips on how to improve one’s gear. Unfortunately, according to users’ opinions, the product has a lot of disadvantages, which results in a final satisfaction rating of 1.9/5.0 [31]. In their solution, only one insole contains electronics; therefore, only one foot is measured. To record a run, the insert has to be calibrated for about 10 min each time. Additionally, according to the users, due to the fact that all the equipment fits inside the insert, they are quite hard and inconvenient. The opinions also include information about insufficient battery capacity and not accurate measurements (the price is about USD 100) [30].

Among the affordable solutions for daily usage, the British company Arion [32] created an insert that contains eight pressure sensors and connects to the phone via Bluetooth. In their mobile application, the following information can be found: the place of the foot with which one presses on the ground, stride length, cadence, balance, contact time, pace, stability, flight time, and muscle load index. Statistics are presented in the app in real-time. The company assumes two training modes, either a user analyzes their recorded results, or they use the application in a trainer mode, where artificial intelligence guides the training. Unlike the previous product, it is necessary only to calibrate the inserts once for 10 min. The only problem that users point out is the nuisance of the application in the event of inadequate running. The application sends information in real-time each time the user is above or below the target range (e.g., step length). Arion offers only four different sizes of inserts, each for the price of approx. USD 400 [32].

## 3. Materials and Methods

In this section, we present the system architecture, hardware design, and data interpretation procedures to provide a clear and comprehensive understanding of the methods and materials used in our system. Moreover, the data flow of the system is discussed, along with the application screen map.

### 3.1. System Architecture

**Figure 2:**
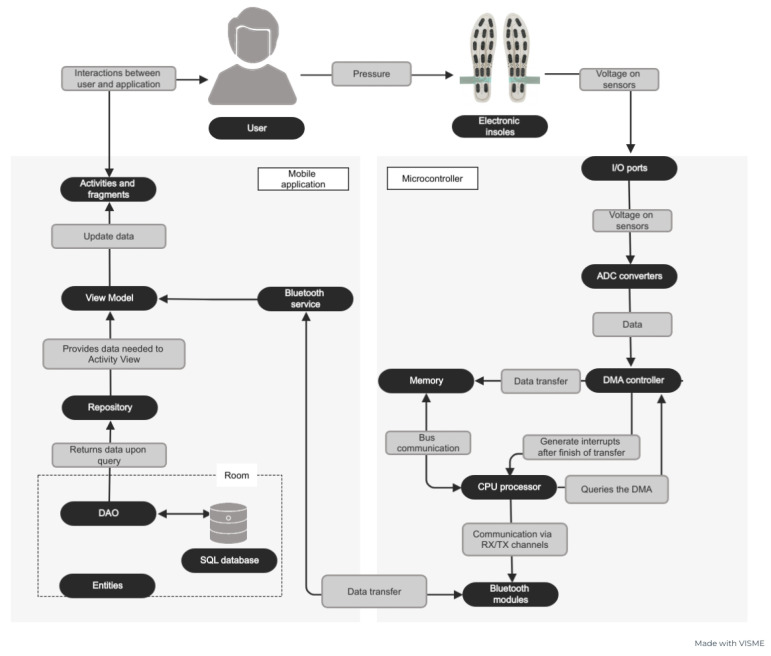
System architecture (View Model—communication center between the user interface and the repository, it simultaneously caches the latest data from the so-called LiveData and passes it to the repository. Repository—manages multiple data sources, in this case, localization services, Bluetooth, and database. Room—the database layer that makes them easier to store, uses DAO (Data Access Object) to query the SQL database. Data Access Object maps SQL queries to functions that can be called in the program. Entities—annotated classes that describe the database table).

Our open-source system was developed based on the requirement specification and the system architecture design. A general design of the system architecture is presented in Figure 2. The detailed requirement specifications and UML diagrams specifying the essential parts of the systems can be found in the Supplementary Materials.

The system requires a one-time setup consisting of turning on the application, providing data such as name and weight, and allowing Bluetooth and location access. Afterward, daily system usage demands only putting on the insoles, turning on the app, and navigating to the tab of the user’s choice.

### 3.2. Hardware Design

**Figure 3:**
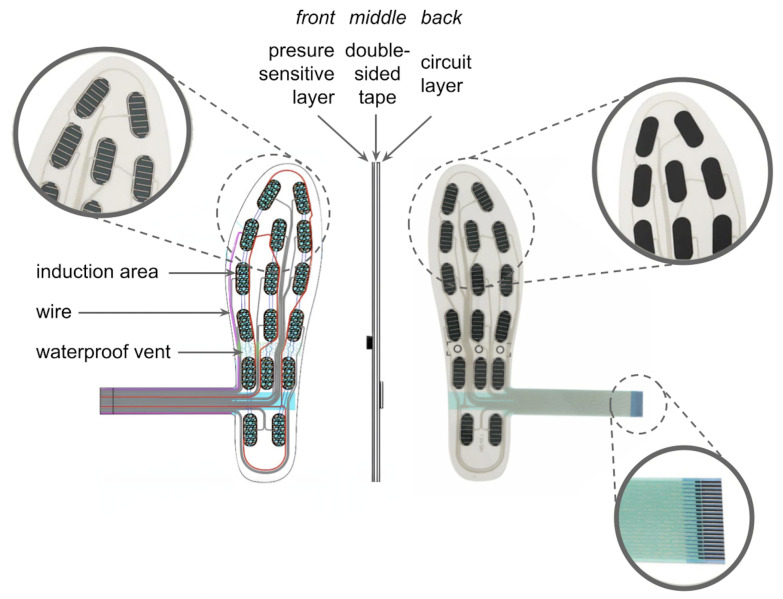
Electronic insole schema.

Our electronic insole is composed of 16 pressure sensors laminated in a transparent film. According to the manufacturer’s description [33], the sensors are triggered by a force of 500 g and are able to measure pressure up to 10 kg. Taking into consideration their number, the measurement range is sufficient. The insoles are thin—about 0.4 mm; they are completely dust resistant and waterproof to short (30-s) immersion in water to a depth of 1 m. Figure 3 presents the insole schema.

The hardware part of the system uses the STM32L010RB microcontroller, which has the following parameters:

- ARM (Advanced RISC Machine) architecture—32-bit chip that gives many more possibilities than 8-bit AVR microcontrollers. In addition, ARM enables the use of multiple ADC channels, etc.;
- ST product—the company provides a microcontroller and appropriate tools for programming, such as STM32CubeMX or STM32HAL libraries;
- CorexM0+ core—the Corex-M family is designed for microcontrollers. “M0+” stands for an improved version of the basic M0 core with reduced power consumption;
- Ultra-Low-Power system—ensures the lowest possible battery consumption and very low power consumption;
- DMA—Direct Memory Access, i.e., direct access to RAM. This module allows unloading the processor by taking care of data transfer between the memory and peripheral systems or between different memory areas.

In Table 2, a few selected parameters of the STM32L010 [34] and Atmega128 [35] processors are compared. These two were selected due to similarities, such as Flash Memory Size.

The above comparison reveals that, although the price of the STM32 microcontroller is comparatively lower, it exhibits superior performance in several parameters as compared to its alternatives.

**Figure 4:**
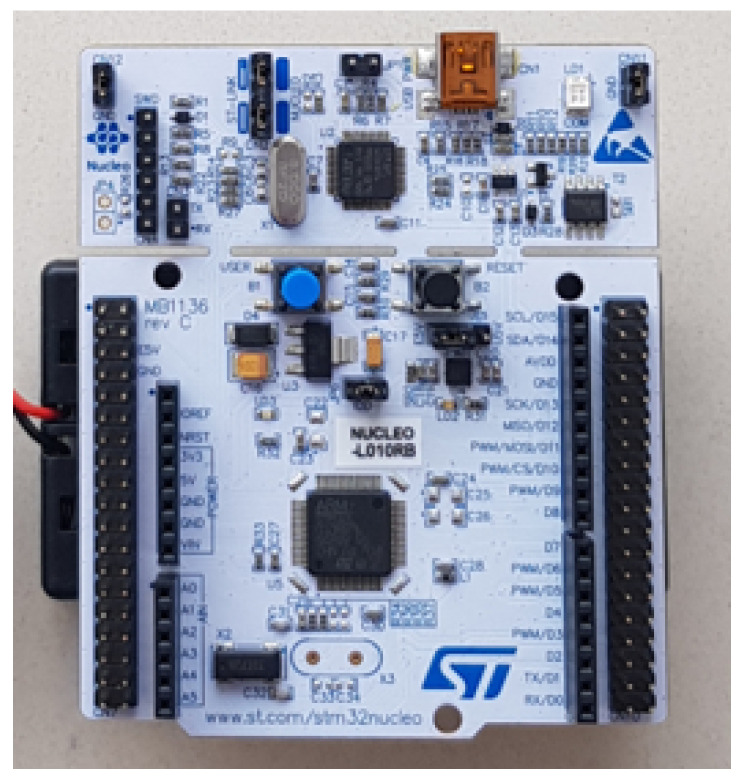
Appearance of Nucleo-L010RB.

STMicroelectronics Development Board—the STM32L010RB microcontroller is located on the development board from the NUCLEO series—STMicroelectronics NUCLEO-L010RB, the appearance of which is presented in Figure 4. Apart from the mentioned microcontroller, the set has a very big advantage—a built-in programmer-debugger ST-Link/v2-1 [34]. It enables quick and convenient programming and testing of created programs. The whole thing can be powered in various ways—including from the battery through the VIN pin and through the USB connector. The 16 ADC channels designed on the board were used to take measurements from 16 pressure sensors. Additionally, the pins of RX and TX are used to enable the connection with the Bluetooth module. It is also possible to break off the part with the debugger to reduce the size of the board, which is important for this project.

**Figure 5:**
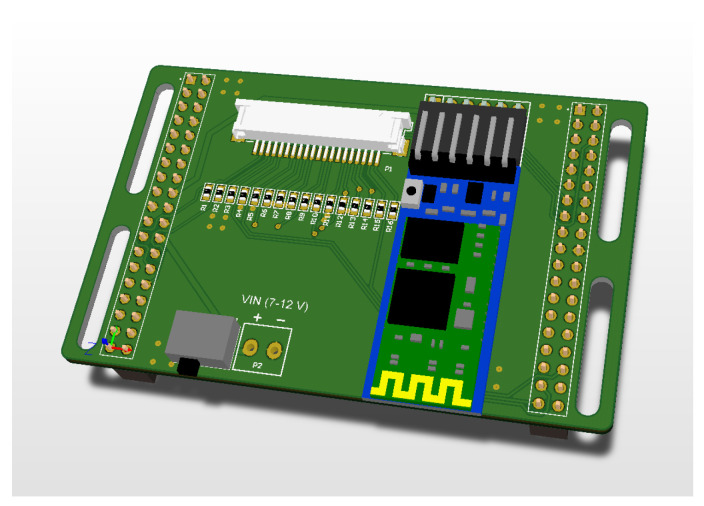
Design of dedicated PCB.

**Figure 6:**
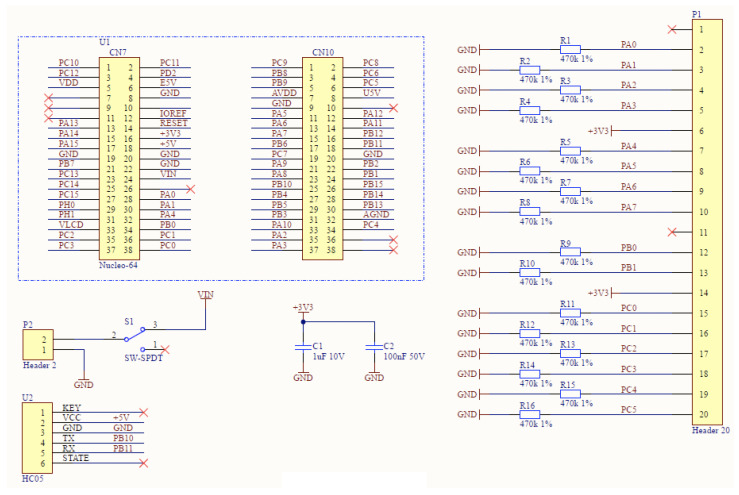
Dedicated PCB schema.

The standalone development board was insufficient for the complete implementation of the system; therefore, additional connectors, switches, cables, and resistors were needed. To avoid a significant increase in the volume of the hardware layer, it was decided to use an additional PCB (Printed Circuit Board) designed specifically for this system. This approach allowed for a more compact and streamlined hardware design while still incorporating all necessary components. Figure 5 presents the layout of the board design, and Figure 6 shows the wiring diagram.

The additional board provides the output of ADC channels to the Molex-type connector, to which the insole is connected. Thanks to this board, a direct connection with the HC-05 Bluetooth module was obtained. There is also a convenient place for the switch and a place to connect the battery. The whole system is enclosed in a quite small size—approx. 70 × 50 mm, which is slightly smaller than the part of the above-mentioned Nucleo development board (after breaking off the debugger part).

The designed PCB includes the following components:

- Bluetooth HC-05 module,
- Molex-type connector, 20 pins,
- SMD 0603 470 kΩ resistors,
- toggle switch,
- female goldpin strip, 8 contacts, angular, single-row,
- two female goldpin strips—20 contacts, straight, double-row.

To configure the peripherals of the STM32 microcontroller, the HAL library and the CubeMX software were utilized, which simplified the use of peripherals, making the code more readable and speeding up programming. The project was created in a free IDE (Integrated Development Environment) in TrueStudio by Attolic See: https://www.st.com/en/development-tools/truestudio.html (accessed on 20 November 2021) [36].

Due to the fact that the selected Bluetooth module is not a Low-Energy module, it was necessary to minimize the power consumption during data transmission in order to preserve battery resources, i.e.:

1. Transmission should take place only after receiving the information about the start of the transmission from the application.
2. Numeric data should be stored in a buffer as small as possible.
3. Data are not transmitted if their maximum read pressure value is less than 10% of the range. Data with the minimum pressure value, i.e., less than 10% of the range, is considered noise. It can appear, for example, as a result of the natural curvature of the inner part of the shoe, its strong tying, etc. During the tests, these minimum values were recorded even when the foot with the sensor did not touch the ground (so we could not talk about any user’s pressure). In conclusion, the transmission of such data was considered an unnecessary use of energy.
4. Transmission takes place at the longest possible time to ensure smooth readings—that is every 150 ms. Measurement every 150ms enables 400 measurements per minute. The maximum running cadence, i.e., the number of steps per minute, is about 170–180 for elite shod runners [37]. According to the Nyquist-Shannon sampling theorem [38], samples should be collected at least twice as often as the measurement period. As mentioned above, the measurements are performed 400 times in a minute, thus at least twice for each, even the fastest, step.

### 3.3. Data Interpretation

The data read from the pressure sensors does not grow linearly. Therefore, it was necessary to map the results to an appropriate scale—for the average user, the easiest scale to read is the percentage scale. In order to properly map the results, a laboratory experiment was carried out. A soft material was placed under the electronic insole, simulating real conditions. A truncated cone, placed with the base with the smaller surface down, was placed in the center of the sensor. Subsequently, further weights were placed on the cone, and the measurements’ values were recorded.

The entire experiment was repeated ten times for each of the six weights. The results of averaging and rounding of the collected measurements are presented in Table 3.

**Figure 7:**
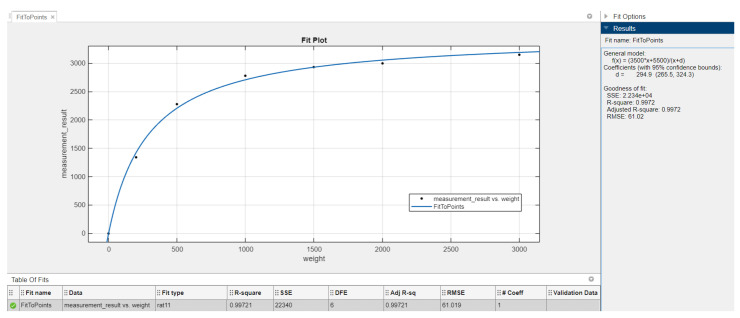
Curve Fitting Tool: Function matching.

Then, the data from Table 3 were divided by 16, yielding a scale of 0–4095 instead of a scale of 0–65,535. This procedure was performed in order to better match functions with the lowest possible degree. The collected data are the points to which the pressure function had to be adjusted. Using Matlab and the Curve Fitting Tool (Figure 7), the best-fitting function with the smallest possible complexity was selected (see Equation (1)):

(1) $$y=\frac{3500x-5500}{x+300}$$

After finding the appropriate function, it was necessary to remap the values so that they indicated the percentage scale. The formula for mapping the values was based on the function presented in Equation (1) and is as follows (see Equation (2)):

(2) $$x\;=\;\frac{-300y-5500}{y-3500}$$

### 3.4. System Data Flow

**Figure 8:**
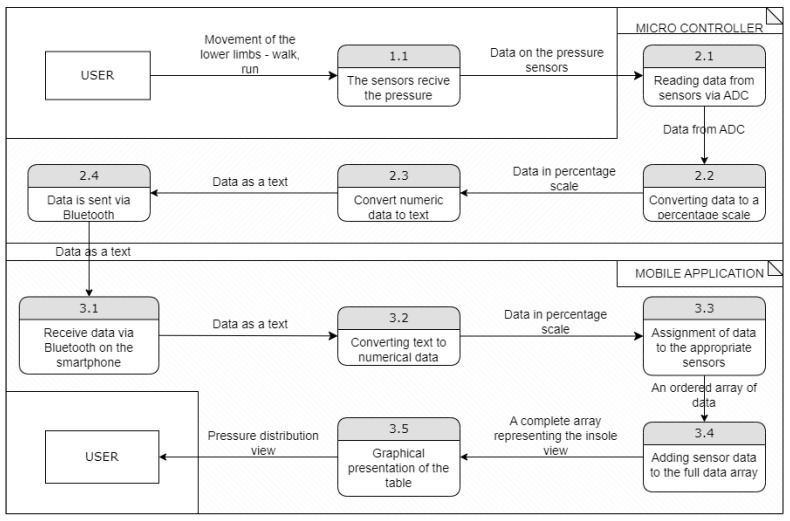
Data flow diagram of data in the system.

The diagram in Figure 8 evinces the data flow in the system. It starts when the user begins to put pressure on the insoles until it is displayed on the smartphone screen, in case the user decides to test the device without saving the parameters to the database.

The STM32L010RB that was used in the project is equipped with analog peripherals—there are sixteen 12-bit ADC channels. The measured voltages on the mentioned microcontroller peripherals get to the memory thanks to ADCs (Analog to Digital Converters). These convert the analog value (voltage) into digital form. This is performed with the help of DMA memory allocation in order to avoid overloading the processor.

The ADC sampling rate in the case of this microcontroller is up to 1.14 Msps. For this project, the ADC Clock is set to 2.097 MHz, and the sampling time is 160.5 cycles, so it is possible to measure signals from ADC channels approximately 12,500 times per second. As a result, the reading of the data takes place almost simultaneously—in fact, they are read at very small intervals (every 0.8 μs), which, in total, gives about 14 μs to read all 16 sensors. Due to the fact that data is sent via Bluetooth every 150 ms, the mentioned 14 μs are negligible.

The data are converted to a percentage scale (0–100%) and then converted to text and sent via Bluetooth. The application stores an array with the configuration of the foot view—it assigns addresses where the sensors are located and where the insole cannot reach.

The mobile app receives the textual data, which are converted to numerical values, organized, and included in the foot view configuration array. Further, based on the above-mentioned array, a graphic is created—a view of the foot pressure distribution presented to the user.

**Figure 9:**
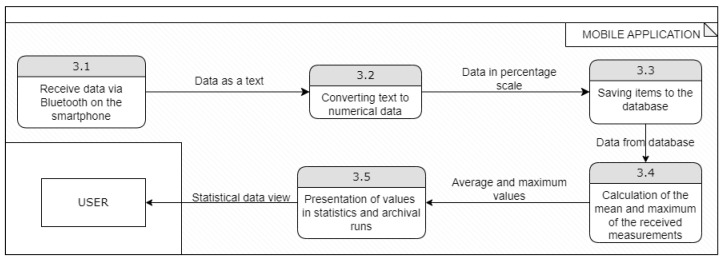
Data flow diagram—workout option.

There is also another possibility—the user runs the application and decides to perform a workout—in this way, the data should be written to the database instead of being presented in a graphical form. The data flow diagram, in this case, will differ only in the part related to the mobile application, as presented in Figure 9.

### 3.5. Application Screen Map

**Figure 10:**
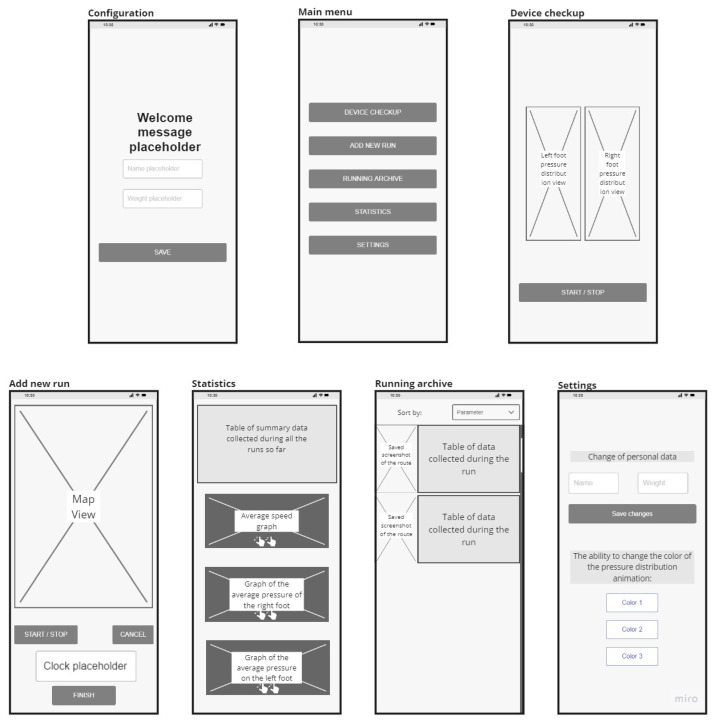
Mobile application screen map (Configuration screen only on the first execution of the app. The Device checkup, Add new run, Statistics, Running archive, and Settings screens are secondary to the Main menu screen).

For designing a mobile application, the user journey was provided using the application screen map presented in Figure 10. The following screens were designed:

- The “Configuration” window appears only on the first use of the application and contains a space for a welcome message, two text fields in which the name and the current weight should be entered, as well as the “SAVE” button.
- “Main Menu”—appears after the configuration is completed, or as the first screen when the application is used again. Each button leads to a selection of one of the five remaining tabs.
- The “Check Device” window provides a view of the pressure distribution for each foot and a button for starting and stopping the generation of subsequent views.
- The “Add New Run” tab consists of a map view, a clock, and three buttons to start and stop a training session, cancel it, or end it saving the workout.
- “Archived Runs” is a screen containing a table with summary data collected from all previous runs. In addition, it can display three charts—the average speed and the average pressure on the right and left feet.
- The “Statistics” window contains a view of all the runs so far—it consists of a view of the route traveled and a table of data collected during the run. At the top of the screen, there is an additional dropbox where a user can select the parameter by which the gears will be sorted.
- Finally, the “Settings” tab contains text fields for a new name or changed weight, the “SAVE” button, and three buttons for selecting the color of the graphics in the “Check Device” tab.

## 4. Results

**Figure 11:**
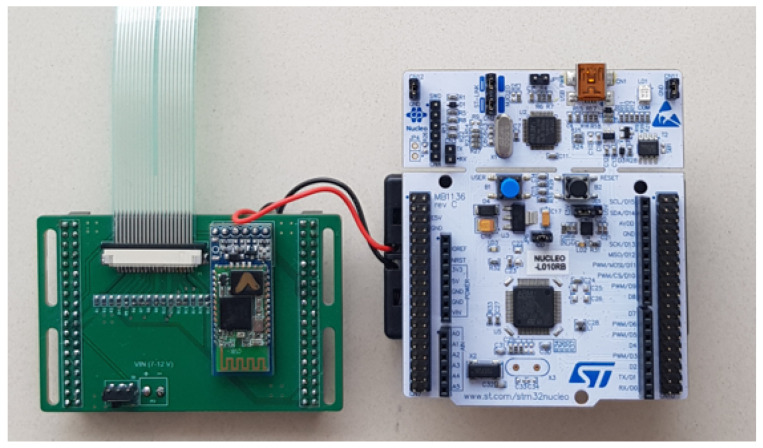
Nucleo L010RB with dedicated PCB.

**Figure 12:**
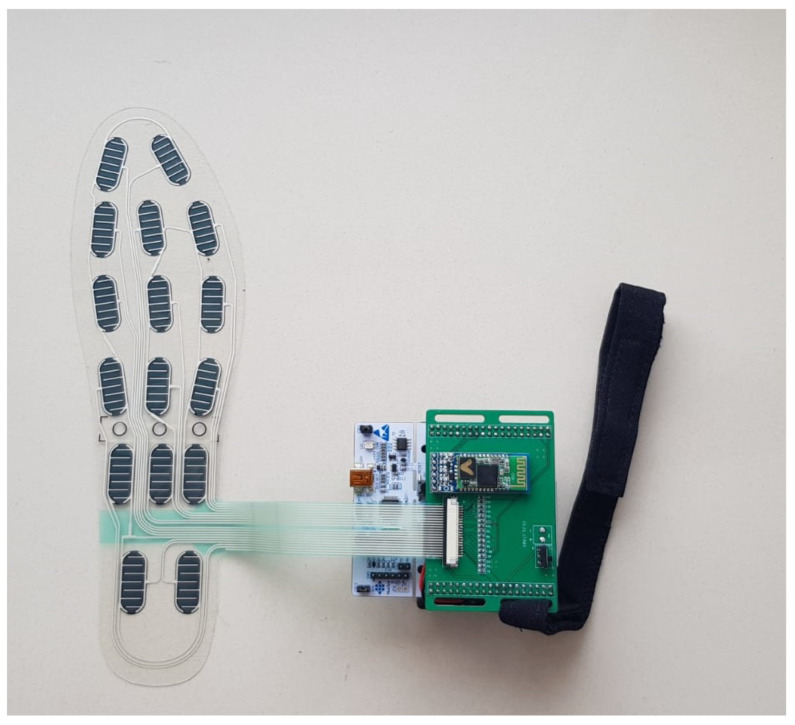
Full hardware presentation.

Based on the PCB presented in the previous section, we developed the hardware part of the system. The Nucleo L010RB integrated with dedicated PCB is presented in Figure 11, and the connection to the insole is presented in Figure 12. The software part was implemented in accordance with the system data flow specification.

The designed application was implemented on the Android system using Android Studio software in Kotlin language, using the Dagger framework and the Hilt library [39].

**Figure 13:**
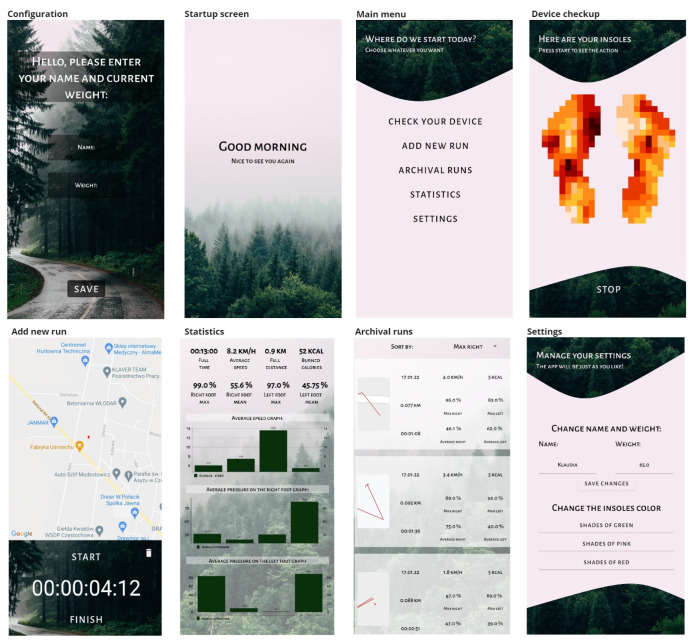
Screenshots of our mobile application.

The application utilizes coroutines, allowing the application to run in the background and implement a working system for receiving data via Bluetooth. Figure 13 shows the final version of our prototype mobile application for monitoring the pressure distribution of runner’s feet and the insole management.

The system validation was performed according to the following protocol:

- operational testing of the Bluetooth module,
- measurement of current consumption,
- verifying the conversion of numeric data to text and vice versa,
- shoe insole graphic presentation,
- operational testing of individual modules of the mobile application.

**Figure 14:**
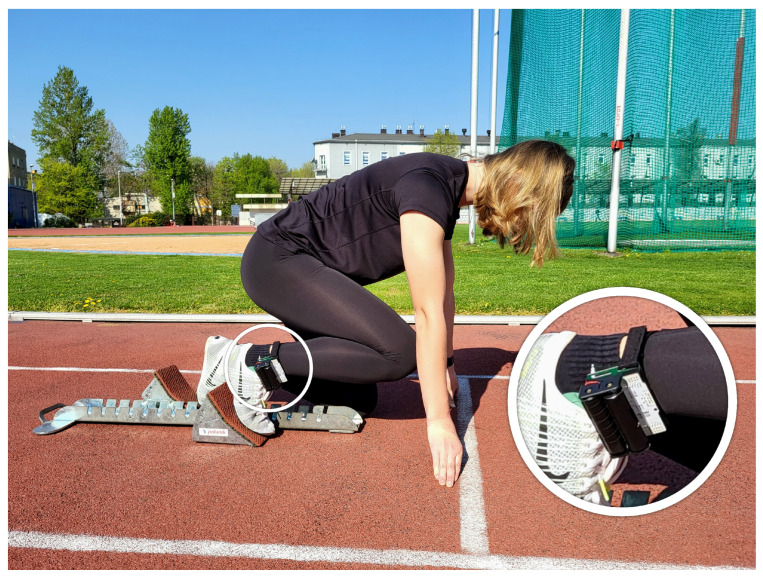
Photo of the hardware part of the system taken during tests.

All system modules were tested according to the testing protocol. Figure 14 presents the hardware part of the system during the tests.

### 4.1. Operational Testing of the Bluetooth Module and Measurement of Current Consumption

The Bluetooth module consumes the most power during pairing attempts—about 80 mA [40]. We used a laboratory current source with a milliamp multimeter, and experimentally verified that during continuous transmission (sending data 400 times per minute), without introducing energy-saving solutions, the Bluetooth module consumes 25 mA. The microprocessor that is offloaded by the use of DMA needs relatively little current—a maximum of 2 mA.

The major design challenge concerning the proper selection of the battery was the input voltage. According to the STM32-Nucleo [41] documentation, the voltage on the VIN channel must be in the range of 7–12 V. The battery voltage decreases with its discharge; when using two 3.7 V batteries connected in series, there is a reserve of 0.4 V left.

It was experimentally verified that the batteries used are sufficient to ensure the functioning of the system. The connected device was left for 12 h to verify energy consumption. During the experiment, the device was programmed to try to pair with another device, which caused three times more energy consumption. This is considered a worst-case test scenario. After the experiment, we measured the voltage in the batteries—7.2 V, which was sufficient to ensure the proper functioning of the system.

**Figure 15:**
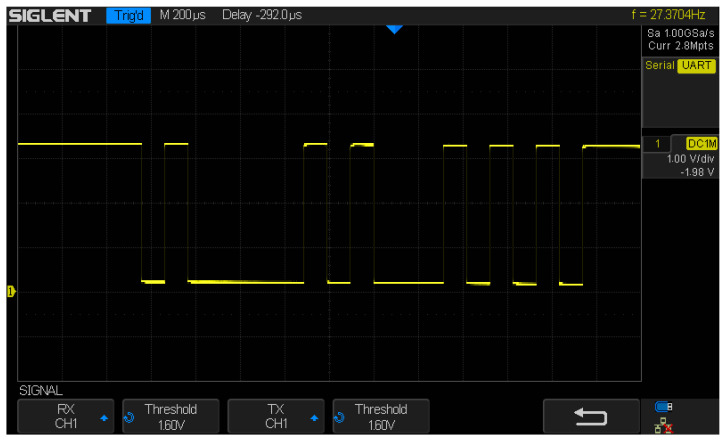
Sending sample value—“AT”—to verify Bluetooth transmission. As it is seen in the screenshot, it is correct—the “AT” binary value is “0100000101010100”.

**Figure 16:**
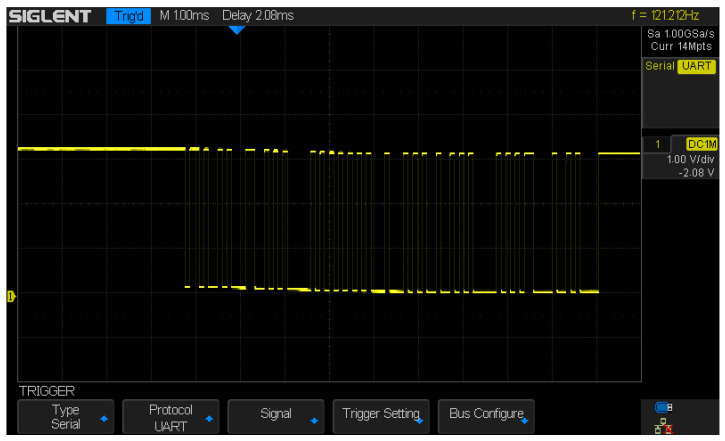
Sending “KLAUDIA”—one more time, verifying Bluetooth transmission, with a longer message.

An experiment using an oscilloscope was conducted to check if the system was sending data via the Bluetooth module. The device was connected to the RX channels and ground (GND), then the test program was launched. The experiment showed the proper Bluetooth transmission, as shown in Figure 15 and Figure 16.

Bluetooth data integrity testing was performed using the Saleae [42] logic analyzer, which was responsible for reading bit data and presenting their values from an ASCII table. The test showed that the data sent by the Bluetooth module is correct—the logic program correctly displayed the words that were encoded in the microcontroller program.

### 4.2. Verifting the Conversion of Numeric Data to Text and Vice Versa

**Figure 17:**
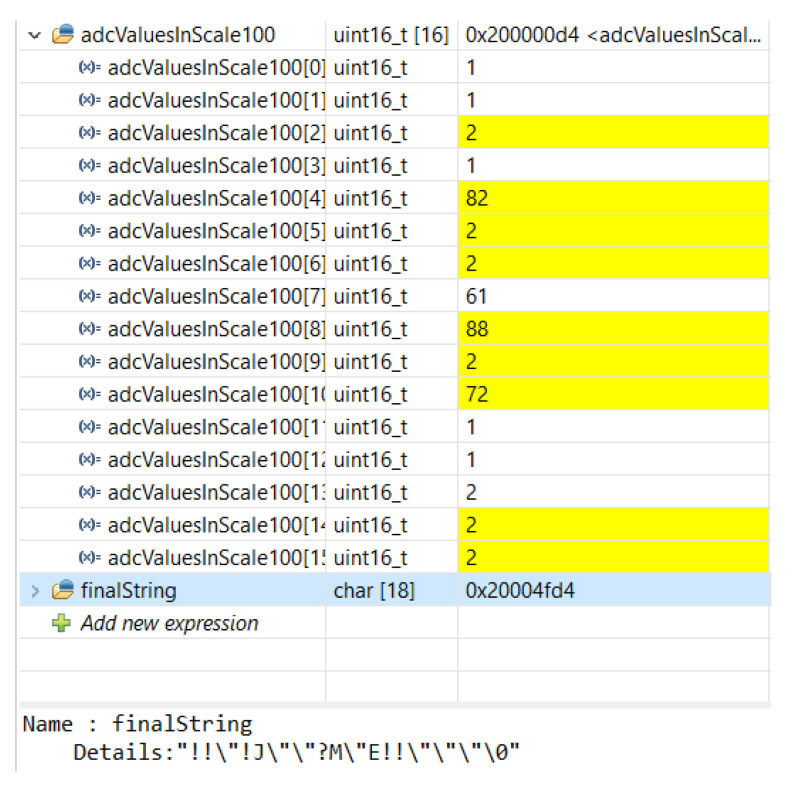
Screenshot of data conversion to char variables.

**Figure 18:**
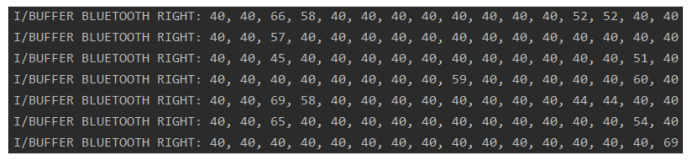
Converting data received via Bluetooth.

**Figure 19:**
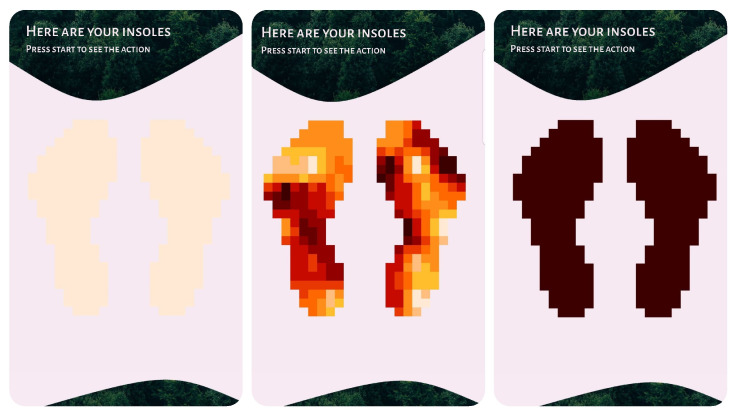
Screenshots from the mobile app with pressure distribution view.

**Figure 20:**
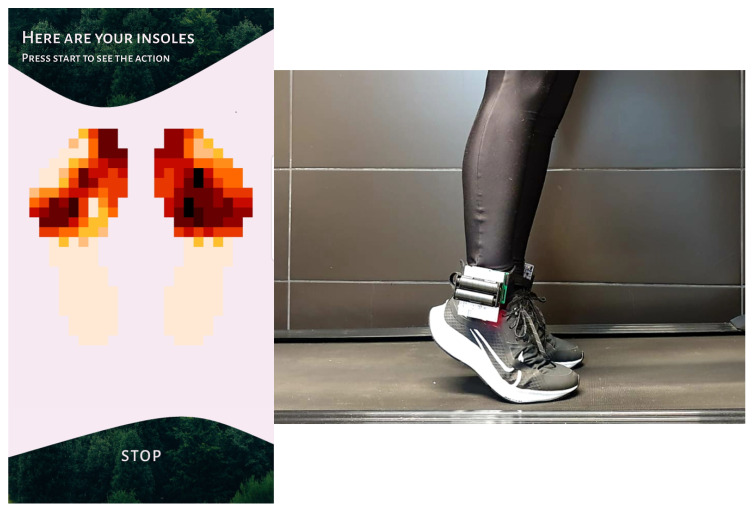
Test example of pressure distribution.

Verifying the conversion of numeric data to text and vice versa consists of the following steps:

1. Low-power operation principle:
  We selected the low-power STML010RB module and developed an algorithm minimizing energy consumption. Data are transmitted via Bluetooth only if the pressure is present. This was verified in the following experiment setup. We changed a random bit to a higher one (imitating the pressure on the sensor), and we observed that the algorithm automatically turns off the sleep mode and transmits the relevant data to the mobile application. The max variable is the maximum value taken from the sensors. If it is less than 10, it means that the highest value is less than 10% of the maximum value—these data can be considered no traffic. The flag_IfTransmit variable is 0 when no Bluetooth transmission has been made and 1 when data have been sent.
2. Data decryption:
  We used the ASCII character encoding system [43]. Data read from ADC channels are numerical data in the range of 0–100%. One array element is 1 bit of data, which enables the transfer of a number between 0 and 255. Therefore, the numeric data were replaced with a char variable, e.g., the number 40 was sent as ’(’. The conversion algorithm was verified by random input numerical data. The adcValuesInScale100 array stores the values taken from the data sensors on a scale of 0–100%. The finalString variable is an array of char variables that are sent by the Bluetooth module to the mobile application. After comparing the data presented in the screenshot (shown in Figure 17) with the ASCII [43] table, we noticed that the data are converted correctly.
3. Mobile device data acquisition and data integrity testing:
  The incoming data integrity was verified by reprogramming the microcontroller and sending generated datasets. The incoming data were compared with the generated datasets. The test revealed that the data are received correctly by the mobile application.
4. Application data decoding:
  The application was tested to ensure that the re-conversion to a number was correct. A test dataset encoded in the microcontroller firmware was used, and performed the algorithm operation in order to generate an array of int variables containing the appropriate numerical values. The performed experiments successfully showed the same data as previously encoded in the microcontroller firmware, which is presented in Figure 18.
5. Foot pressure distribution graph:
  Another important feature is displaying the foot pressure distribution. The resolution of the visualization is 10 × 22 squares. Based on the arrangement of sensors on the electronic insert, the source locations were selected (each of them is marked as 2 squares due to the shape of the pressure sensors). The data received via Bluetooth was mapped to the appropriate addresses. In this way, the first 32 fields of the table were completed. In order to fill the remaining fields, a function was implemented that calculates the average of the eight nearest neighbors for each empty field. We tested the correctness of this revealed by providing a different sample input. The test has shown that the method is correct. The effect can be seen in Figure 19). The test example pressure distribution is presented in Figure 20.

### 4.3. Users’ Evaluation Protocol

We designed the following evaluation protocol for the entire system. Ten Android users (five women and five men in the age range of 15–30 years) were asked to install the application on their smartphones and test the operation of the application. Users used different versions of Android software and phones with different screen sizes. After 20 min of testing, each user was asked to complete a short system performance evaluation survey. They were to rate the individual aspects of the system on a scale of 1–5, where 1 meant “very bad” and 5 “very good”. The aspects evaluated by our test users were: Wearing comfort, Bluetooth performance, Measurement accuracy, App appearance, App performance, and Willingness to continue using the app. The collective results are presented in Table 4, while additional user comments are summarized below.

After analyzing the results, we have discovered the following:

- Wearing comfort—users rated the comfort of the shoe insole highly, which is thin and practically imperceptible while running. The major drawback of the system is its weight due to its large batteries.
- Bluetooth performance—users had no objection to this part of the system.
- Measurement accuracy—users were asked to perform exercises such as: standing on one leg, jumping, standing on tiptoes, etc. Users reported that the view of the pressure distribution presented in the application is consistent with reality.
- Application appearance—another topic that the users had to comment on was the view of the mobile application—this part of the system was assessed very positively. Even users with a smaller screen size did not mention any GUI issues.
- Application performance—users evaluated the operation and stability of the mobile application. All tests were performed on smartphones with Android version 9 and newer. Users reported no problems with launching and using the application.
- Willingness to continue using the application—the majority of users would choose to continue using the device for health monitoring purposes. The only inconvenience reported was the size of the device and the battery.

Taking into account all the above-mentioned information, the presented system prototype can be considered fully operational and meets all the assumptions, including the functional and non-functional requirements specified at the beginning.

## 5. Discussion

Although the presented solution is not supported by any commercial organization, there are several advantages of our project. In comparison with other systems, our device provides a long working time without charging (min. 72 h), while Digitsole [30] and Arion [32] offered only 8 and 10 h, respectively, or Mustufa’s et al. solution [20], which provided only 120 min of work. There is no need for device calibration as it works properly from the moment one turns it on—in contrast to many other solutions, such as Arion and Digitsole, as shown in [16,23,24,25].

Our device has a large number of sensors, which translates into a much more accurate measurement of pressure distribution, so the device makes it possible not only to compare the power of pressure between the two feet but, above all, to pay attention to where the pressure is greater in the foot. Our solution is equipped with 16 sensors, while Jung et al. [19], Jagos et al. [21], Roth et al. [22]. Chadel et al. [28] created solutions with a very limited number of 4–6 sensors. Thanks to the frequent pressure measurements, 400 times per minute (every 150 ms), our solution allows for the correct reading of maximum pressure values, which are recorded for a very short time. Moreover, the placement of electronics on the outside of the shoe is much more favorable to users than placing them in the insole itself.

Wireless connection to the phone provides full mobility of the device; thus, this enables one to use it anywhere and anytime. In addition, it is possible to use the application in the absence of an Internet connection, as well as its operation in the background. It is worth mentioning that there are solutions without wireless data transmission—for example, Sorrentino et al. [16], Lin et al. [17].

On the other hand, with the Internet connection, thanks to real-time data processing, it is possible for a trainer, physiotherapist, medical practitioner, or user to analyze the image instantly at any time and place. GPS tracking helps to track workout results and keeps the user motivated to stay active with the device.

Finally, the cost of the device is much lower than the other solutions—we estimate the cost of the whole system at 150 EUR, while there are systems that cost up to 250 EUR (Arion [32]), 500 EUR (Sorrentino et al. [16]), or even USD 1000 (around 950 EUR), Saito et al. [15]).

Table 5 presents the comparison of our project to the two most similar commercial solutions. It is extremely important to emphasize that our solution, however, is built in the open-source model that encourages enhancements and open collaboration.

There are several limitations of our solution at its current stage of development. It requires a permanent connection to the phone via Bluetooth; it lacks the analysis of the risk of injury and the assessment of the run quality. The size of the entire device is large due to shortages in the availability of microcontrollers on the market. For improvement, all the hardware should be replaced by a single small PCB connected to the insole. The software, in such case, would not need to be changed. Another disadvantage of our system is that it does not provide any AI for the quality of the run assessment, which could prompt a user on how to improve the running performance. Therefore, this will be investigated in our future works.

## 6. Conclusions

This paper described a novel open-source solution that allowed for the construction and development of the strain gauge system for monitoring runner’s feet pressure distribution. In our research, we analyzed the market for pressure monitoring insoles, designed a suitable hardware system, and developed the appropriate microcontroller software with particular attention to low power consumption. The Bluetooth communication algorithm was implemented, and a method for displaying graphics representing pressure distribution was developed.

The design requirements have been met, and various aspects of our system have been tested for its evaluation.

Moreover, the mobile application was also implemented and tested on an emulator available in the Android Studio software as well as a real device. The main advantages and novelties of our solution are:

- the device provides a long working time without charging (min. 72 h),
- there is no need for device calibration,
- the device contains a large number of sensors, which means a more accurate measurement of pressure distribution (it allows comparison of pressure between the feet, but also the distribution of pressure in a foot),
- it allows correct reading of maximum pressure values, which are recorded for a very short time (thanks to frequent pressure measurements every 150 ms),
- the placement of electronics on the outside of a shoe is more favorable to users than placing it in the insole itself,
- wireless connection to the phone provides full mobility of the device,
- the application can be used in the absence of an Internet connection, as well as operate in the background,
- thanks to real-time data processing, it is possible for the trainer, physiotherapist, or user to analyze the image instantly at any time and place,
- GPS tracking helps to track workout results and keeps the user motivated to stay active with the device,
- the cost of the device is much lower than other solutions,
- and last but not least, it is in an open-source model, which encourages enhancements and open collaboration.

Our system is still being extended and enhanced. The currently planned works include minimizing the hardware components (by replacing all hardware components with a single, small PCB with a microcontroller and Bluetooth Low-Energy module, reducing the power source, and adding a memory card) and allowing the data to be stored on a memory card in case there is no Bluetooth pairing device available. Moreover, we are also planning to add new software features, such as the possibility to track and compare pressure from archived workouts, integration with other apps (such as Google Fit or Samsung Health) and devices (smartwatches, etc.). As the system can be easily further developed, our future works will also focus on better algorithms that would provide real-time information for the users running unevenly, too fast, or taking too many steps. Eventually, we would also like to explore the usability issues of our device by focusing on its comfort side. This can be performed either by minimizing the device or integrating sensors directly into shoes.

## Figures and Tables

**Table 2:** Comparison of parameters of Atmega128 and STM32L010RB.

	Atmega 128	STM32L010
PRICE	about USD 15.25	about USD 4.11
ARCHITECTURE	8 Bit	32 Bit
CLOCK	16 MHz	32 MHz
MEMORY	128 KB FLASH	128 KB FLASH
RAM	4 K × 8	20 K × 8
DMA	none	present

**Table 3:** Table of measurements after averaging the values.

Number of Measurement	The Weight [g]	Read Value
1	200	21,440
2	500	36,480
3	1000	44,480
4	1500	46,880
5	2000	48,000
6	3000	50,400

**Table 4:** Results of system tests (scale 1–5, where 1—very bad, and 5—very good).

No.	Wearing Comfort	Bluetooth Performance	Measurement Accuracy	App Appearance	App Performance	Willingness to Use It
1	4	5	5	5	5	5
2	4	5	4	5	4	5
3	3	5	4	4	5	3
4	5	5	5	5	4	5
5	4	4	5	5	5	5
6	3	5	5	5	5	4
7	4	5	5	5	5	5
8	4	5	5	4	4	5
9	4	5	4	5	4	4
10	4	5	5	5	5	5
Avg	3.9	4.9	4.7	4.8	4.6	4.6
Avg	4.58					

**Table 5:** Comparison of the effects of work with commercial solutions.

Product Name	Digitsole [30,31]	Arion [32]	This Project
User rating (0–5 scale)	1.9 [31]	no data	4.58
Price	100 EUR	250 EUR	150 EUR
Working time	8 h	10 h	minimum 72 h
GPS tracking	yes	yes	yes
Necessity of calibration	10 min each time	10 min only once	no need
Quality of run evaluation	yes	yes	no
Amount of sensors	no data	8	16
Sensor types	no data	accelerometer, gyroscope, pressure sensors	pressure sensors
Electronics placement	in one of the insoles	on the outside of both shoes	on the outside of both shoes
Collecting data without a database connection	yes	no	no
Injury risk data analysis	yes	yes	no
Open source project	no	no	yes

## Data Availability

The code for the implementation of the system for the research presented in this paper is openly available online in the GitHub code repository: https://github.com/KlaudiaKromolowska/Foot_plantar_pressure_monitoring_system (accessed on 29 December 2022).

## Supplementary Materials

The following supporting information containing the system requirement specification and system design can be downloaded at: https://www.mdpi.com/article/10.3390/s23042323/s1.
