# Supplementary material for: Open-Source Strain Gauge System for Monitoring Pressure Distribution of Runner’s Feet
Source: Sensors (Basel). 2023 Feb 19;23(4):2323. doi: 10.3390/s23042323 (PMC9959378; doi:10.3390/s23042323)
Supplement: Supplementary file 1 [file sensors-23-02323-s001.zip › sensors-2159912-supplementary.pdf]

Supplementary materials to the paper: "Open-source strain gauge system for monitoring pressure distribution of runner's feet"

Klaudia Kromołowska, Krzysztof Kluza,
Eliasz Kańtoch, and Piotr Sulikowski

List of Tables

1 General functional requirements for the system 2
2 Detailed functional requirements for the mobile application 3
3 Non-functional requirements for the system 4

List of Figures

1 Use case diagram 5
2 The mobile app design – UML activity diagram 7
3 The mobile app design – UML class diagram 8
4 The UML sequence diagram for "Checking the device" 9
5 The UML sequence diagram for "Adding a new run" 10

1 System Requirements and Design

The development of our system started with the specification of its users. Subsequently, we gathered the descriptions of major processes occurring in the system. Based on that, we elicited the requirements to be met by the system, and defined the system architecture.

Our system will require a one-time setup consisting of turning on the application, providing data such as name and weight, and allowing Bluetooth and location access. Afterward, daily system usage will demand only putting on the insoles, turning on the app, and navigating to the tab of the user's choice.

1.1 System Requirements

Our system should enable the analysis of the distribution of foot pressure forces. Thus, it is possible to distinguish some basic functionalities of the system, such as: receiving input data by the microcontroller, sending data via Bluetooth to the application, data processing, saving them to the database, and graphical presentation of the results. The lists of functional requirements of the whole system and the mobile application are presented in Tables 1 and 2, respectively. Table 3 provides a description of the non-functional requirements, focusing primarily on how the system works, its security, speed, etc.

**L.p.**	**Requirement description**
1	Reading the measurement from the strain gauge sensor
2	Reading multiple measurements simultaneously
3	Sending data downloaded from the microcontroller via Bluetooth
4	Real-time data transmission
5	A mobile application that can be installed on Android phones
7	Calculation of the pressure function based on the data received by the microcontroller
8	Presentation of the pressure measurement results as a percentage scale
9	Display the view of the inserts on the phone
10	Record data from pressure sensors collected during training
11	Ability to run the application in the background
12	Ability to use the application without internet access

Table S1: General functional requirements for the system

L.p.	Requirement description
1	Start and stop the pressure animation
2	Add a new training (workout) session, stop it, resume it, save it or cancel it
3	Track your route on Google maps during the training
4	Record the parameters of each training session, such as route, workout duration, average speed, distance traveled, and the average and maximum pressure exerted by each foot
5	View parameters saved in previous runs
6	Ability to delete a saved run
7	Ability to sort saved runs according to the selected parameter
8	Ability to change the color of the insole animation, and the user information
9	Ability to stop and resume the run from the notification bar

Table S2: Detailed functional requirements for the mobile application

After defining the requirements, another important element allowing the creation of a complete system model is planning the architecture of the entire system. It should primarily concern the structure, mutual interactions of the subsystems, and functionality of the system and its possible interactions with the user. In order to better illustrate that, a use case diagram (Fig. 1) was prepared.

In our system, two subsystems can be distinguished – the part related to the microcontroller and the other one section with the mobile application. The process commences with the user who exerts some pressure on the electronic insoles and wants to read how that pressure is distributed over the different parts of each foot in the mobile application.

Strain gauge sensors placed in the inserts are deformed under pressure, thus changing their resistance. As a result, specific analog values appear on the appropriate ports of the microcontroller, which are converted into digital form by ADC (Analog to Digital Converter) converters. DMA (Direct Memory Access) controls the data transfer and transfers the data to the memory, offloading the load from the processor – thus acting as a direct link between the ADCs and the memory. The CPU (Central Processing Unit) reads data from memory and transmits it to the Bluetooth module using RX/TX (Receive/Transmit) channels. The Bluetooth module sends the data, which is then intercepted by the Bluetooth service in the mobile application.

The data from the Bluetooth service is transferred to the View Model to the

**Security**	The microcontroller should only send data via Bluetooth to the paired device. The mobile device will not send data to other devices, thus avoiding additional risk.
**Availability**	The system should be available whenever the user has access to a smartphone with a Bluetooth module and a device with insoles.
**Utility**	The system is created for the needs of athletes and amateurs who want to take care of their health by monitoring the distribution of foot pressure on the ground.
**Speed**	The application should be launched within 3 seconds. Data display should be refreshed at least 400 times per minute.
**Ease of use**	The application requires a clear user interface. Aesthetics and simplicity of use are important – the application should be operated in an intuitive manner.
**Compatibility**	Due to the fact that this is the first version (prototype) of our application, there will be no need to care about backward compatibility. The design should be made in a way that allows easy editing of the system and written in the Kotlin language for better clarity.
**Completeness**	The system will be considered complete if it is able to retrieve data, transform it and display it in a mobile application.
**Compatibility**	The application should work fully on Android smartphones with version 9.0 and be convenient for use on screens ranging between 5 and 6.5 inches in size.

Table S3: Non-functional requirements for the system

so-called LiveData, or data managers are used to observe changes in the data and update the view. The rest of the data needed for the view should be retrieved from the repository. The repository queries the database using the methods defined in DAO to get the relevant data. After loading the appropriate data, the data from the repository is transferred to the View Model. Activities or portions of the user interface respond to changes in the View Model and update the view, thereby responding to user interaction.

The next part focuses on use cases to illustrate the key functionalities of the system that are visible from the user's point of view. As presented in the diagram (Figure 1), there are five basic use cases. The following list contains both the names of the use cases and the related functionalities:

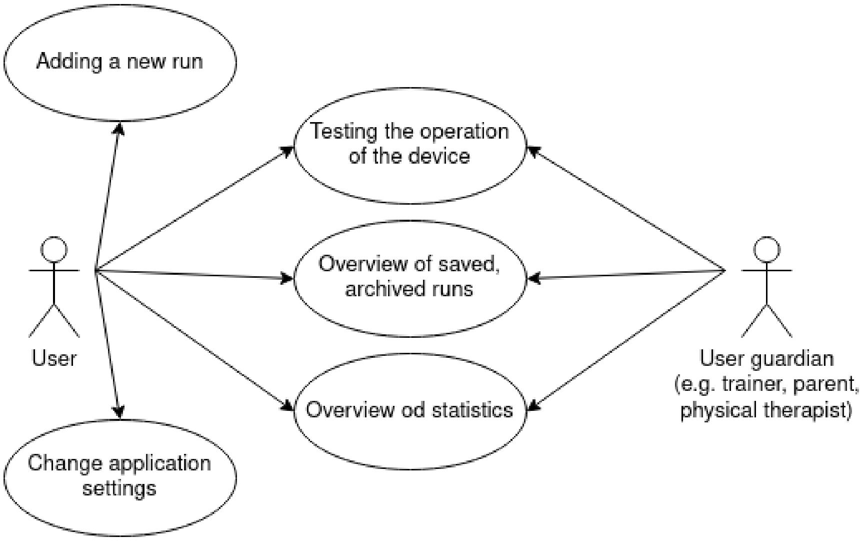

Figure S1: Use case diagram

1. *Testing the operation of the device* – the user wants to check how the device works or how the pressure of the feet is distributed on the surface of the insoles. The user can start and stop the pressure distribution visualization at any time. It is also possible for this part of the application to be used by the user's guardian (e.g., a parent, coach or physiotherapist) while the user is using the device.

2. *Add New Run* – the user wants to add a new run. When the "START" button is pressed, the location tracking begins and the timer shown on the screen starts. The user can pause the measurements at any time, cancel the run or terminate it, thus saving it to the database.

3. *Review Archived Runs* – the user wants to review their previous workouts. The user has the ability to sort the runs by a selected criterion, as well as delete each run. This part of the application is also available for the user's guardian, who can view the achievements of the user according to the selected criterion.

4. *Statistics overview* – when the user or the guardian wants to see a summary of the training so far. The user can see summarized parameters from all runs, such as the total number of kilometers run and graphs showing the average speed and pressure of each foot in successive runs. The user can zoom in or out of the graph view with a gesture to see the details.

5. *Change application settings* – the user wants to personalize the application. It is possible to change the color of the presentation displayed in the tab related to testing the device, as well as to change the personal data previously saved.

The operation of the system is ensured by the following implementation elements:

- an implementation of microcontroller software to obtain data from the sensors and send it over Bluetooth,
- an implementation of an algorithm to scale the data from 0-65535 to 0-100,
- an implementation of an Android mobile application,
- an implementation of an algorithm responsible for visualizing the collected data.

In the following diagrams, we provide a detailed description of the software design. The UML activity diagram in Fig. 2 reveals the steps performed by the mobile application for the designed system. If the mobile app is launched for the first time, it will ask for the user's name and current weight and check if they are correct. If the application does not have permission to retrieve the location or connect via Bluetooth, a dialog box will be displayed to inform the user and perform further steps. The standard menu of the application has five options: "Checking the device", "Adding a new run", "Overview of Archived Runs", "Overview of Statistics", and "Settings".

Since the app is developed in the object-oriented paradigm, it allows for easy modifications and extensions. The UML class diagram of our mobile application is presented in Fig. 3. The **ConfigurationFragment** class is responsible for the first launch of the application. The **MainActivity** class is the main menu view that leads to the selection from the five available tabs: **InsolesView** (that controls the **RightFootView** and **LeftFootView**), **TrackingView** (that uses **TrackingService**), **RunsView** (contains an instance of the **RunsViewMode** class, and is associated with other running module classes), **StatisticsView**, and **SettingsView**.

The behavior of our software system is presented using the UML sequence diagram. It focuses on the two most relevant and characteristic use cases for our project – "Checking the device" (Fig. 4) and "Adding a new run" (Fig. 5).

All auxiliary functions are replaced by the "Application Logic" object for simplicity. There are four objects for testing the operation of the device: user, GUI, application logic, and Bluetooth controller. In the case of the "Adding a new run" diagram, two additional objects are distinguished: the GPS (Global Positioning System) service and the database.

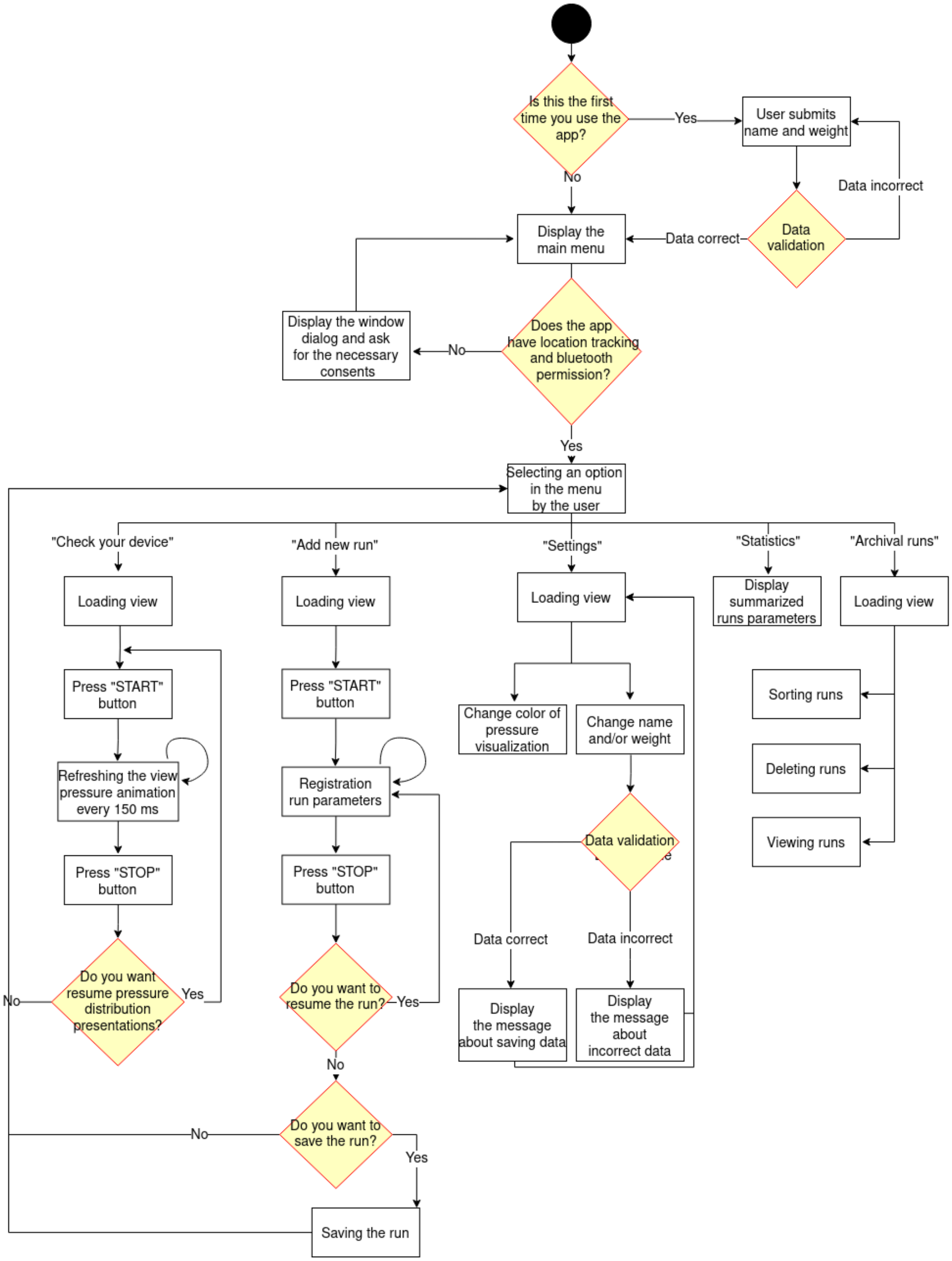

Figure S2: The mobile app design – UML activity diagram 7

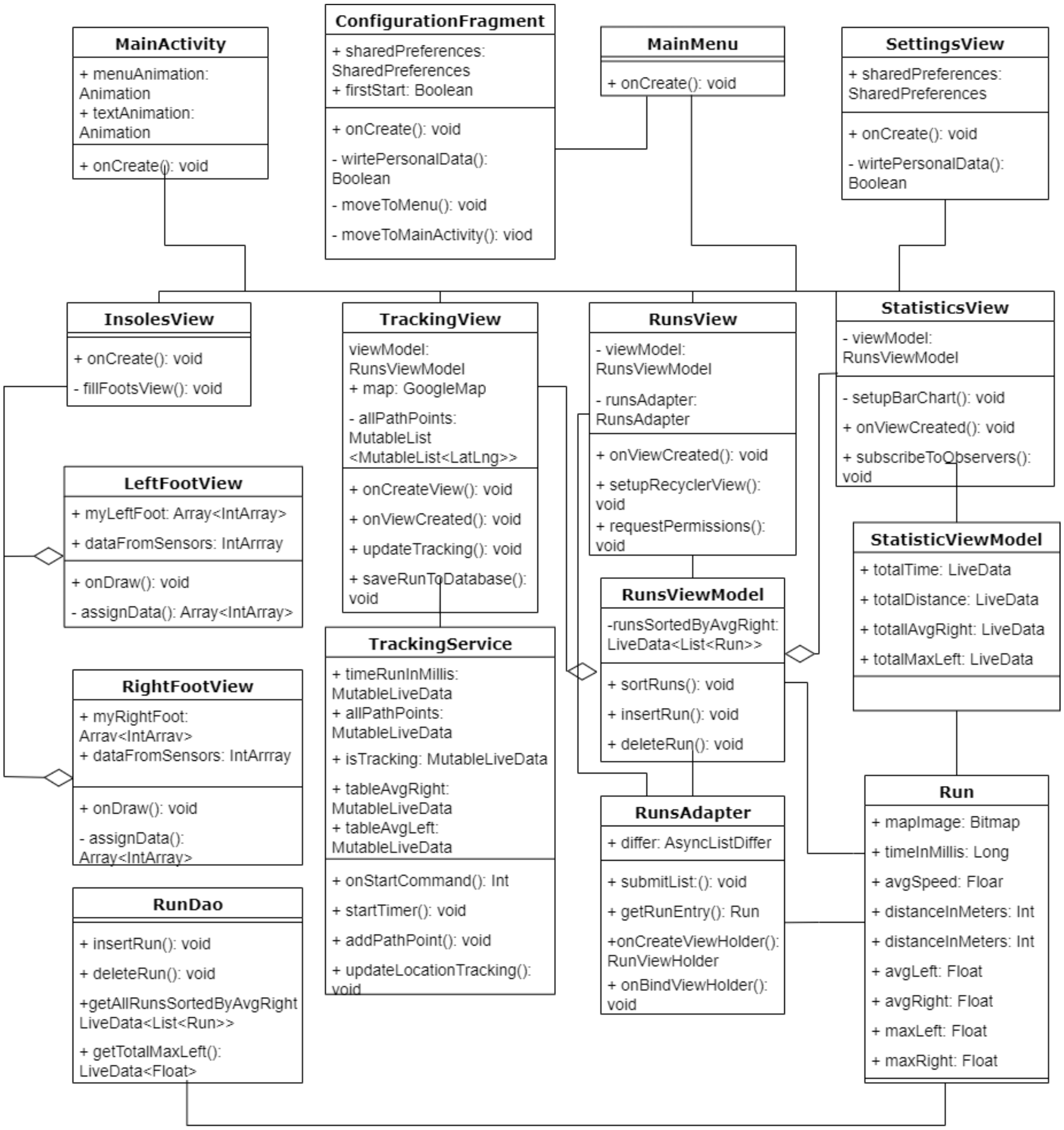

Figure S3: The mobile app design – UML class diagram

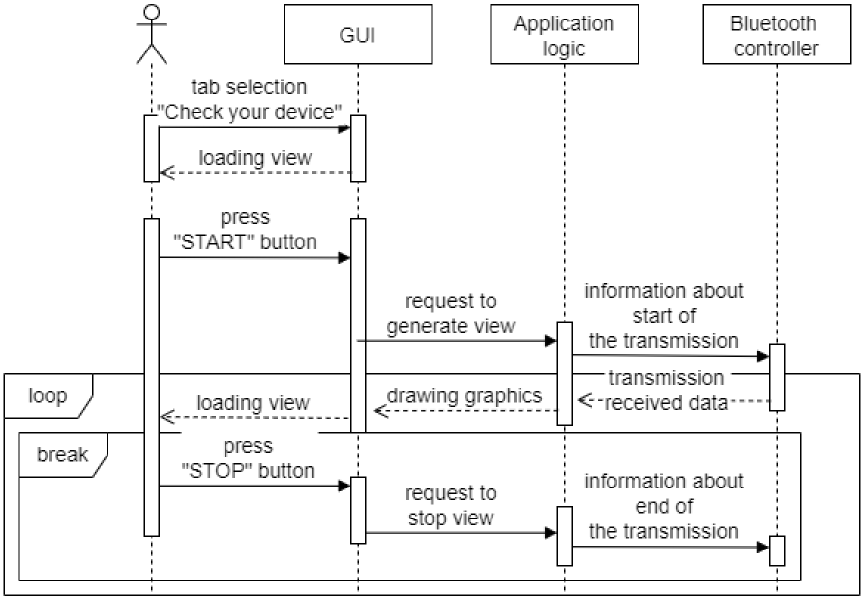

Figure S4: The UML sequence diagram for "Checking the device"

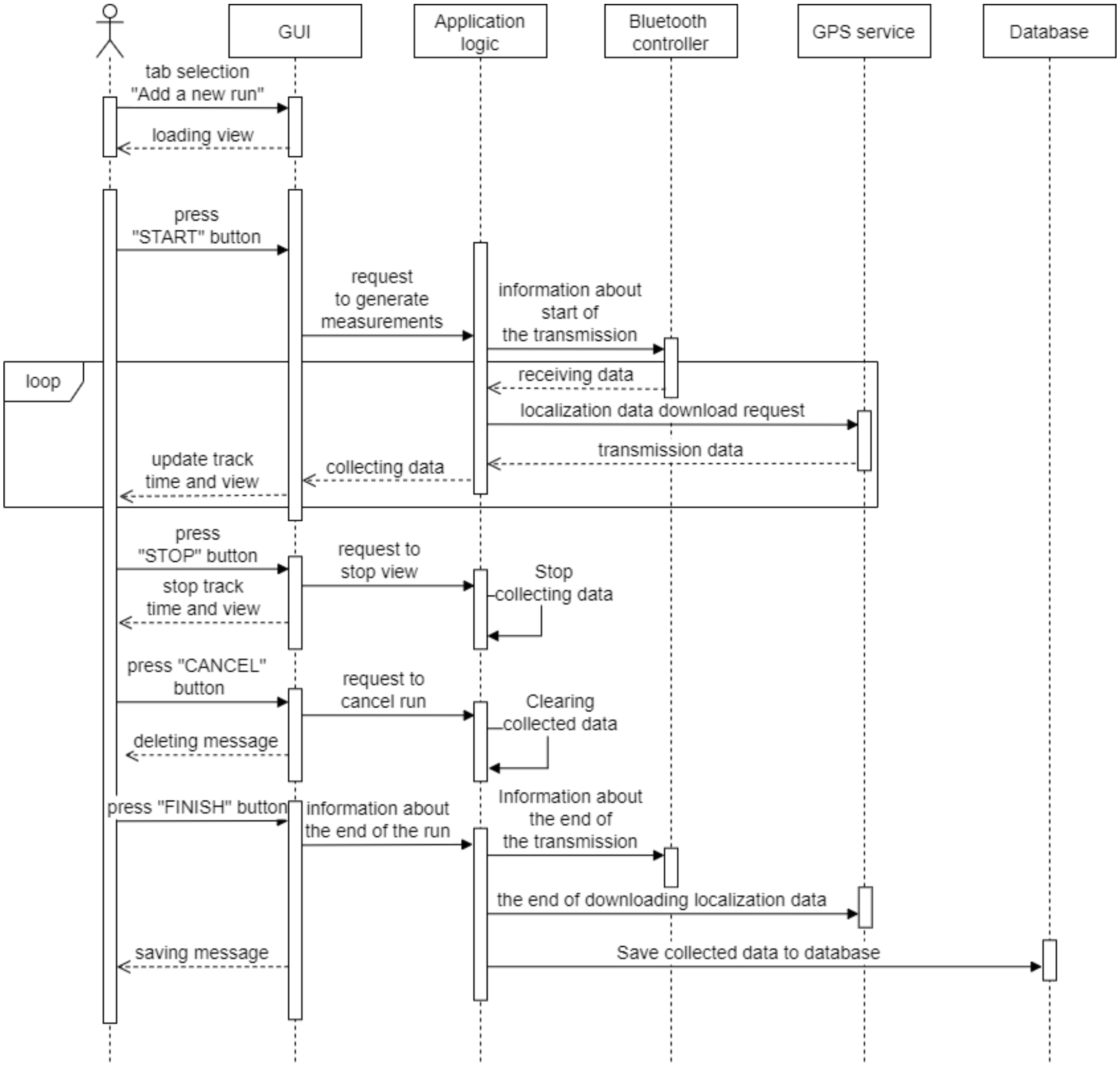

Figure S5: The UML sequence diagram for "Adding a new run"
